# New findings on SNP variants of human protein L-isoaspartyl methyltransferase that affect catalytic activity, thermal stability, and aggregation

**DOI:** 10.1371/journal.pone.0198266

**Published:** 2018-06-01

**Authors:** Jeungjin Kim, Baihe Chen, Jean-Louis Bru, Eric Huynh, Mahsa Momen, Dana W. Aswad

**Affiliations:** Department of Molecular Biology and Biochemistry, University of California Irvine, Irvine, California, United States of America; Shiraz University, ISLAMIC REPUBLIC OF IRAN

## Abstract

Protein L-isoaspartyl methyltransferase (PIMT/PCMT1), a product of the *pcmt1* gene, catalyzes repair of abnormal L-isoaspartyl linkages in age-damaged proteins. *Pcmt1* knockout mice exhibit a profound neuropathology and die 30–60 days postnatal from an epileptic seizure. Here we characterize four new SNP variants of human PIMT with respect to enzymatic activity, thermal stability, and propensity to aggregation. Under standard assay conditions, L191S, A150V, P174H and A65V showed activity losses of 72%, 64%, 61%, and 11% respectively. By differential scanning fluorimetry, melting temperature deviations were -5.2, -4.5, +0.5, and -3.4°C. SDS-PAGE of purified protein reveal significant aggregation of L191S, A150V, and P174H, but not A65V. We also report new data on three unusual PIMT variants among the 13 recently characterized by our laboratory. A7P and I58V were previously found to have 1.8–2.0 times the activity of WT PIMT in the standard assay; however, upon kinetic analysis, we find both variants exhibit reduced catalytic efficiency (Vmax/Km) due to weak isoaspartyl substrate binding. The near complete loss of activity (<1%) seen in R36C was investigated by comparing activity of two artificial variants. R36K shows 4.6X the activity of R36C, while R36A shows no improvement, suggesting the guanidino nitrogens of the R36 play a key role in binding the methyl donor S-adenosyl-L-methionine (AdoMet). The new findings reported here extend the list of human PIMT variants that may contribute to neurological diseases in the young and the decline of CNS function in the aged.

## Introduction

The protein repair enzyme protein L-isoaspartyl methyltransferase (PIMT; product of the *pcmt1* gene) plays a critical role in maintenance of cellular protein integrity, especially in the central nervous system [1–3]. A complete loss of PIMT activity in knockout (KO) mice results in altered behavioral patterns, reduced cognitive function, abnormal hippocampal neuron distribution and electrophysiology, and ultimately death by epileptic seizure at 30 to 60 days [4–7]. PIMT heterozygous (HZ) mice, having a 45–50% reduction in enzyme activity, accumulate isoaspartyl-damaged proteins at 3 to 6 times the rate of wild-type (WT) mice [8], and male HZ mice have a reduced life span [9]. More importantly, in two separate studies, PIMT activity levels have been linked to human lifespan and the quality of aging [10, 11]. As of this writing, there are at least 42 relatively rare missense mutations in the coding region of the human *pcmt1* gene.

To evaluate the possible role of rare PIMT mutations in neurological disease and aging, it is fruitful to know how such mutations affect the function and stability of this enzyme. Toward this goal, we recently expressed and characterized 13 rare SNP variants of PIMT (a 226 amino acid protein) that were known at the time [12]. In our standard assay, three of these were found to have reduced activity relative to WT: R36C (<1%), G175R (< 60%), and F72L (~ 70%). G175R also showed a high propensity to aggregation, while R17H was highly thermo-labile. Surprisingly, two mutants (A7P and I58V) had methyltransferase activity in the standard assay nearly twice that of the WT. Two damage predictions algorithms (SIFT and fathmm) were found to correlate well with our findings.

Since that work was published, the number of reported SNPs for human PIMT has roughly tripled. Due to the limited resources of our laboratory, it was not feasible for us to express and characterize all of these new variants. Therefore, we used three SNP-effect algorithms, all based in part on tolerance to single amino acid variations, to select four new variants with a high probability of functional damage. As we show here, all four of these SNPs have significant damage with regard to enzyme activity, thermal stability, or propensity to aggregation. We also present new information regarding the three most unusual SNPs from our previous study; R36C (with barely measurable PIMT activity), and two other SNPs (A7P and I58V) that had methyltransferase activity in our standard essay nearly double that of WT PIMT.

## Materials and methods

### Expression and purification of PIMT variants

Custom expression plasmids for all PIMT variants were based on the pET28a(+) vector and were purchased from GenScript (Piscataway, NJ). DNA coding for the human WT PIMT sequence (*pcmt1* gene) was synthesized with codon optimization for expression in *E*. *coli*, and inserted between the BamH1 and Xho1 sites. Plasmids for SNP variants were generated from the parent WT plasmid using PCR, and the integrity of all plasmid inserts was verified by DNA sequencing.

Expression and purification of PIMT variants was carried out in our laboratory using *E*. *coli* BL21(DE3) as described previously (20). The purified WT PIMT had a specific activity of ~16,000 pmol/min/mg using γ-globulin as the methyl-acceptor (27). Proteins were stored at -80 °C, at concentrations ranging from 0.3–1.2 mg/ml in buffer consisting of 25 mM Tris-Cl, pH 7.0, 150 mM NaCl, 5 mM 2-mercaptoethanol, and 10% w/v glycerol. Protein concentrations were determined in triplicate by a microtiter plate version of the Bradford assay (31) with reagent and protocol from Bio-Rad Labs (Hercules, CA), and bovine serum albumin as the standard.

### Electrophoresis and Western blotting

SDS-PAGE was carried out on 1 mm NOVEX NuPAGE 4–12% gels using the MES running buffer (Thermo-Fisher, Waltham, MA). Gels were stained with 0.05% Coomassie Blue R-250. For Western blotting, un-stained proteins were transferred to a PVDF membrane (0.45 μm) that was subsequently blocked with 5% non-fat milk, and probed with a 1/10,000 dilution of HRP-conjugated goat anti-T7 antibody (Bethyl Labs, Montgomery, TX). Immunostaining as carried out with TMB (3,3',5,5'-tetramethylbenzidine) using the 1-Step™ TMB-Blotting Substrate reagent from Thermo-Fisher. PageRuler™pre-stained protein standards were also from Thermo-Fisher.

### PIMT activity assays

**Fig 1:**
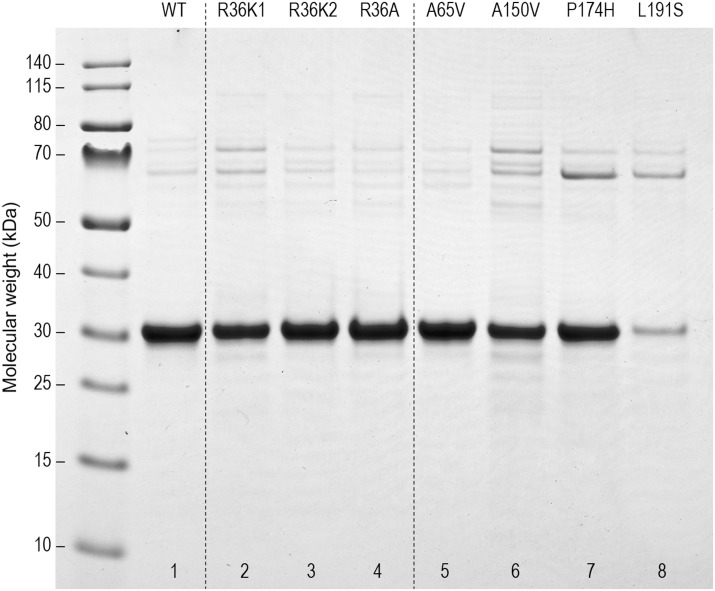
SDS-PAGE of the 8 purified recombinant PIMT variants used in this study. Lanes 1–7 were loaded with 5 μg of protein, while lane 8 had 1 μg due to its low recovery from expression and purification. Stable PIMT aggregates (50–80 kDa) are seen to varying degrees in all lanes, but are most prominent in lanes 2 and 6–8.

PIMT methyl transferase activity was measured in triplicate samples under initial rate conditions using bovine γ-globulin as the methyl acceptor. Prior to each set of assays, PIMT samples (in reaction buffer plus γ-globulin) were split into two groups. One group was subjected to a pre-heat at 52 °C for 15 min prior to the assay, while the other group remained on ice. Assays were carried out in a final volume of 50 μl containing (1) 100 mM BisTris-Cl (pH 6.4), (2) 5.0 mg/ml bovine γ-globulin (from a stock of 25 mg/ml in 10 mM HCl), (3) 0.05 μg of PIMT, and (4) 10 μM S-adenosyl-[*methyl*- ^3^H]L-methionine (^3^H-AdoMet, 10,000 dpm/pmol). Components 1–3 were combined and preincubated in a water bath at 30 °C for 2.0 min. The ^3^H-AdoMet was added, and the reaction was allowed to proceed for 10.0 min, followed by the addition of 750 μl of ice-cold 7% w/v trichloroacetic acid (TCA) to terminate the reaction and precipitate the protein. Pellets were recovered by centrifugation, washed once with 800 μl water and then dissolved in 100 μl of 0.2 M NaOH containing 2% v/v MeOH. 50 μl of this solution was quickly transferred to folded filter paper lodged in the cap of a 4 dram glass vial pre-filled with 2.5 ml of Liquiscint counting fluid, and the vial was then rapidly capped. After incubation at 40 °C for 1 h to allow hydrolysis of the protein methyl esters and transfer of ^3^H-methanol to the scintillation fluid, the cap was replaced and the vial counted by liquid scintillation. The protein values used in the specific activity calculations refer only to the fraction of protein that runs at 28–30 kDa. This was determined by integrating the protein stain in each lane of Fig 1.

New to the methods in this study is the use of isoAsp-DSIP (Bachem Americas; cat. # H-2545) to determine *Km* and *Vmax* values for three previously characterized variants, A7P, I58V, and F72L. Initial rate assays for isoAsp-DSIP methylation followed Johnson and Aswad [13] with modifications. 50 μl reactions were carried out as above, except that (1) the *γ*-globulin was replaced with varying amounts of isoAsp-DSIP, and (2) there was no TCA precipitation step. The ^3^H-AdoMet used in these assays was reduced to dryness in a vacuum centrifuge, and reconstituted in buffer prior to use. This greatly reduces the background level of volatile ^3^H that accumulates in aqueous solutions of ^3^H-AdoMet.

### Differential scanning fluorimetry (DSF)

The thermal stability of PIMT variants was determined using an Mx3005P qPCR machine (Agilent Technologies). Each 40-μL sample contained 20 μl of PIMT (0.29–0.58 mg/ml in storage buffer, pH 7.0) and 40 μM Sypro Orange dye. Fluorescence (excitation and emission wavelengths of 492 and 610 nm, respectively) was recorded over the range of 25–95 °C with a temperature gradient of 1 °C/min. All variants were tested in triplicate on the same plate, and all edge wells were avoided. Tm values were extracted as the inflection point of the fluorescence curves.

### SNP-effect algorithms

SNP-effect algorithms were accessed on-line as follows: SIFT, sift.bii.a-star.edu.sg/ [14]; fathmm, fathmm.biocompute.org.uk/inherited.html [15]; PolyPhen-2, genetics.bwh.harvard.edu/pph2/ [16]. For fathmm, we selected the “unweighted” option.

## Results

### Expression of new PIMT variants

The four new variants examined in this study are listed as the top entries of Table 1, along with 13 of the previously characterized variants. Fig 1 shows an SDS-PAGE gel of the new variants (lanes 5–8), along with two artificial variants at Arg-36 (lanes 2–4) that we expressed to investigate why R36C in our previous study has less than 1% of WT activity.

**Table 1:** PIMT missense variants explored in the current and previous studies.

				Damage predictions		
SNP ID	SNP	Source	PosX^a^	SIFT	fathmm	PP-2^b^
**Current study**						
rs747319067	A65V	ExAC	124	0.00	-4.59	1.00
rs867357446	A150V	dbSNP	209	0.08	-4.23	0.78
rs749892859	P174H	ExAC	233	0.04	-5.40	0.54
rs866455689	L191S	dbSNP	250	0.01	-3.10	1.00
**Previous study**						
rs11540603	A1V^c^	dbSNP	60			
rs200097071	A7P	dbSNP	66	0.14	-2.38	0.47
rs971101823	R17H	dbSNP	76	0.12	-2.81	0.68
rs11540602	A33S	dbSNP	92	0.53	-1.09	0.00
rs374534669	R36C	GO-ESP	95	0.00	-9.18	1.00
rs11540596	H38N	dbSNP	97	0.32	-1.96	0.00
rs143477308	I58V	GO-ESP	117	0.31	-2.91	1.00
rs148018752	F72L	ExAC	131	0.37	0.38	0.00
rs186472718	A79G	1000G	138	0.33	-2.22	0.00
rs4816	V119I	ExAC	178	0.36	0.04	0.00
rs200029629	K124R	ExAC	183	0.31	-0.96	0.01
rs370117092	P162S	ExAC	221	0.37	-1.42	0.01
rs376806476	G175R	ExAC	234	0.06	-7.57	1.00

Colors indicate probability that the mutation is damaging: red (high), tan (moderate), clear (low), as predicted by the indicated on-line algorithm.

^a^ Amino acid position as numbered in the source database. These are all off by +59 from the actual position because they assume an unused transcription initiation codon in the gene.

^b^ PolyPhen-2.

^c^ SIFT warns that damage predictions for A1V are unreliable due to a low number of comparison sequences, so no predictions are listed.

**Fig 2:**
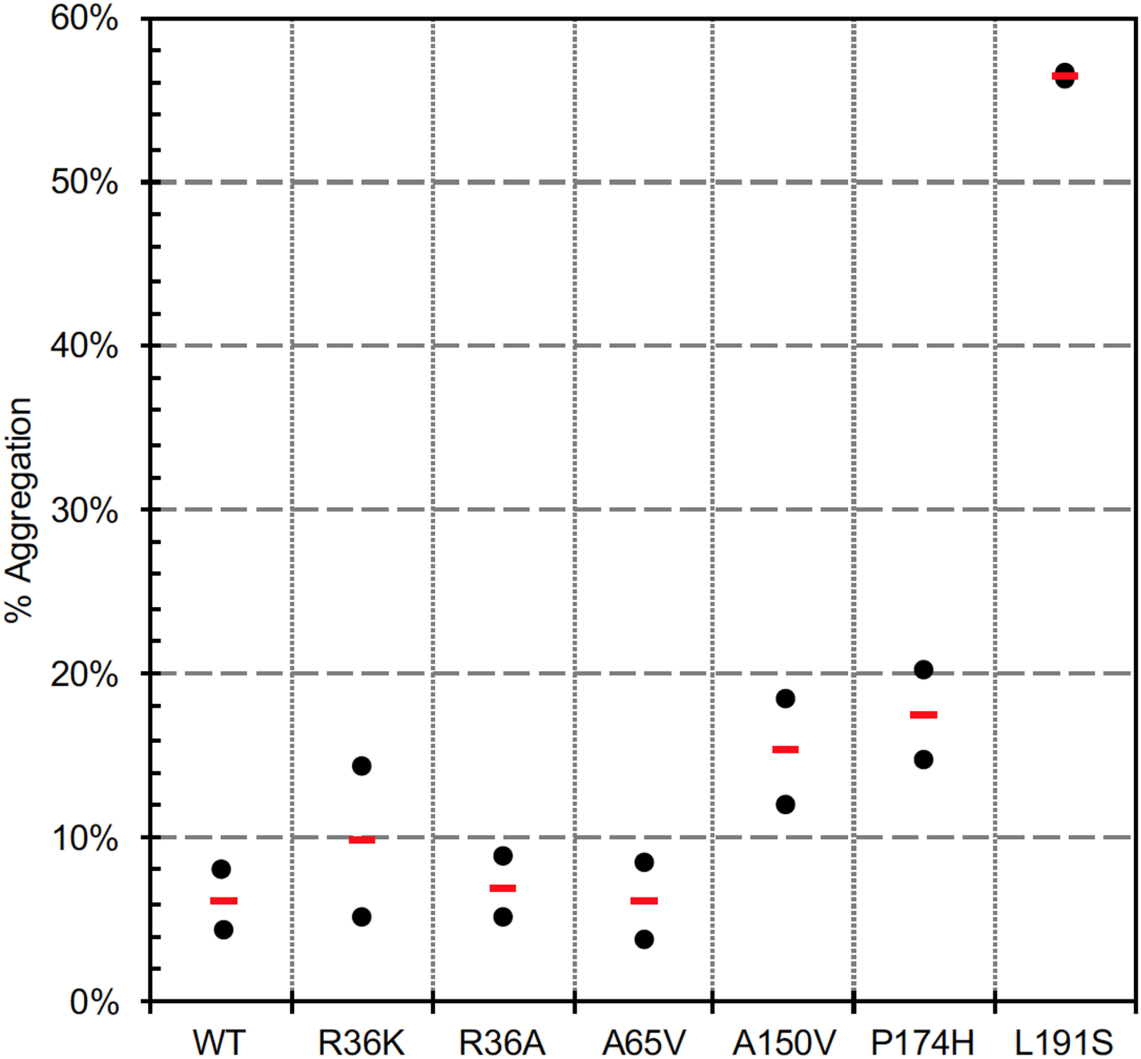
Assessment of variant aggregation. Photos of two stained gels, like that in Fig 1, were analyzed in ImageJ using the lane-plotting function with background subtraction. For each lane, we measured the total peak area in the 30–80 kDa range (P50.80), and the PIMT peak area at 30 kDa (P30). Percent aggregation was then calculated as P50.80/(P50.80 + P30). Data for individual gels is shown with black symbols, and averages with red bars.

Fig 1 suggests A150V, P174H, and L191S are significantly more prone to aggregation than WT or A65V. This is evident from the banding pattern in the 50–80 kDa region, a pattern virtually identical to that seen with several of the previously characterized PIMT variants. These bands were found in the previous study to contain the T7 tag unique to the expressed protein, indicating that the higher mass bands are not due to contamination by *E*. *coli* host proteins. The relative aggregation of all variants shown in Fig 1 was estimated by running two similar gels and employing the lane-plotting feature of ImageJ (imagej.nih.gov/ij/). As seen in Fig 2, aggregation levels are about 15% for A150V and P174H, and 58% for L191S. The pattern of protein staining for L191S (lane 8) is remarkably like that of G175R shown in our previous study [12]. The multiplicity of higher mass bands may reflect structural heterogeneity of the aggregates and/or proteolysis of the largest aggregates.

### Enzymatic activity

**Fig 3:**
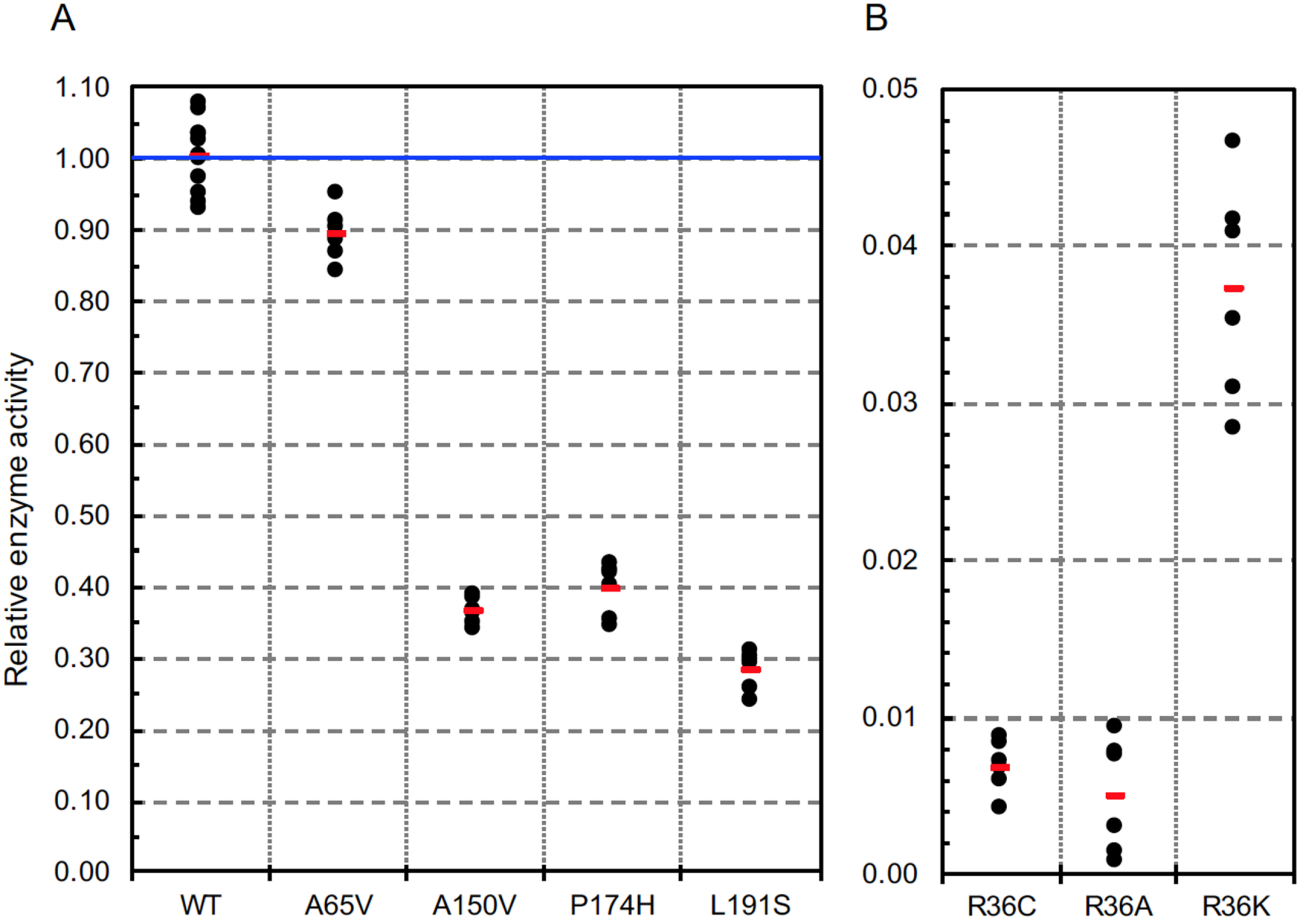
Relative enzyme activities using 5 mg/ml bovine γ-globulin as the methyl acceptor. Assays for each variant were carried out twice, each time in triplicate. Triplicates of the WT enzyme were always assayed in parallel with each variant to provide optimal normalization. Each data point (●) shown in the figure was normalized by dividing the ^3^H-methyl cpm obtained in that assay by the average of triplicate WT assays done in parallel. Red bars indicate the average of the variant data points. Although not always evident due to symbol overlap, there are six data points for each mutant, and 12 for the WT. In panel B, note the vertical scale is greatly amplified due to the very low activity of all three R36 variants.

Results of our standard assay of enzyme specific activity are shown in Fig 3. This assay measures initial rates of methylation of a high concentration (5 mg/ml) of bovine γ-globulin, and should therefore be considered as an estimate of *Vmax* for this substrate. A65V shows only minor impairment, losing about 10% activity compared to WT, while the other three natural variants show more extreme activity losses of 60% or greater (panel A).

Panel B of Fig 3 shows how the artificial variants R36A and R36K compare to R36C, the natural mutant previously shown to have less than 1% of WT activity. We anticipated the R36K artificial variant would restore at least partial activity. This proved to be the case, providing a 5-fold enhancement over R36C, albeit the increase was modest in absolute terms, reaching only 3.5–4.0% of WT. The activity of R36A was the same or worse than R36C.

### Thermal stability

**Fig 4:**
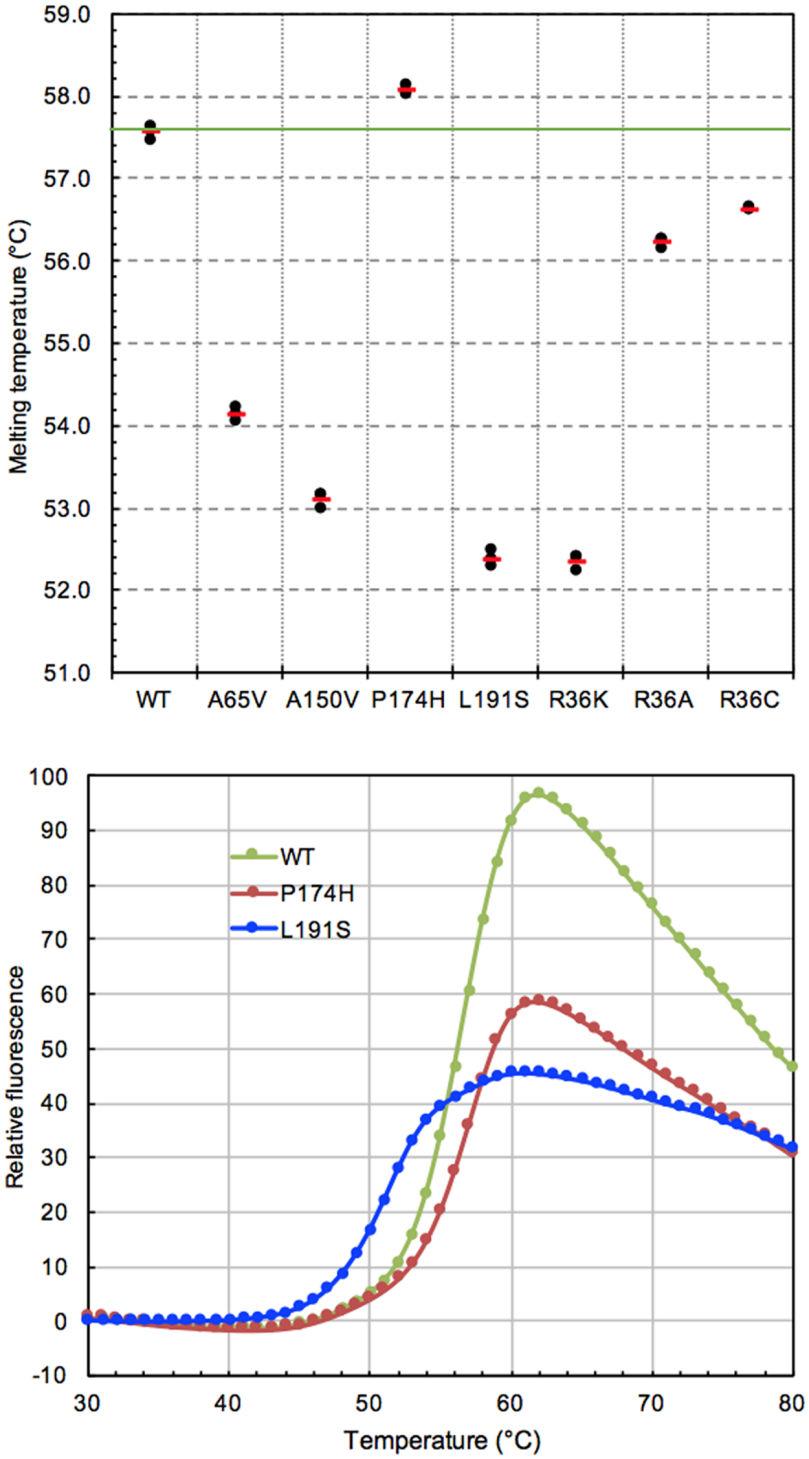
Thermal stability of new variants compared to WT as measured by DSF. Triplicate samples of all variants were subjected to DSF in the same multi-well plate, using conditions described previously [12]. Melting temperatures (black symbols in top panel) were taken as the point where the second derivative of the melting curves (as exemplified in bottom panel) equaled zero. Red bars show average values. Melting temperature for the WT (57.6 ± 0.1 °C) seen here is essentially the same as our previous WT measurement (57.7 ± 0.2 °C).

As in our previous study, we used differential scanning fluorimetry (DSF) to measure the thermal stability of all the new variants (Fig 4). Three of the natural variants, A65V, A150V, and L191S, had significantly lower thermal stability than WT as indicated by melting temperature decreases of ≥ 3°C. Interestingly, P174H appears slightly more stable than WT, similar to the very common variant V119I. All three R36 variants showed decreased stability, but, surprisingly, R36K was by far the most affected, even though it is the most conservative substitution.

### Kinetic constants for methylation of isoAsp-DSIP

**Fig 5:**
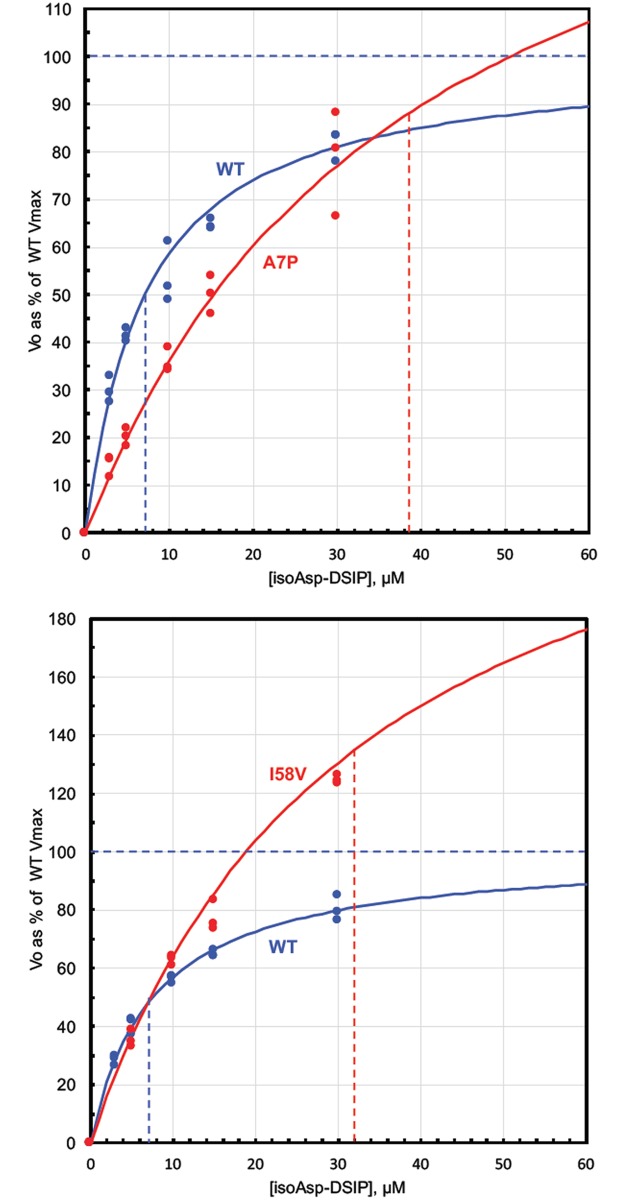
Determination of kinetic constants for variants A7P and I58V using isoAsp-DSIP as methyl acceptor. All assays were carried out in triplicate as described in Materials and methods. For WT (both panels) and A7P (top panel), isoAsp-DSIP was varied over the range of 3–30 μM. For I58V (bottom panel), isoAsp-DSIP was varied over the range of 5–100 μM, but data points for 100 μM are not shown. Initial rates measured at each concentration of isoAsp-DSIP are superimposed on a curve defined by the Michaelis-Menten equation using values of Vmax and Km calculated via the Direct Linear Plot method of Eisenthal and Cornish-Bowden [17, 18]. The colored vertical dashed lines intersect the X-axis at the corresponding value of the calculated Km for isoAsp-DSIP.

We previously observed that variants A7P and I58V have specific activities nearly twice that of WT. As mentioned above, the standard assay approximates *Vmax* values, and not enzyme efficiency (*Vmax/Km*). Enzyme efficiency is especially relevant to PIMT function *in vivo* because it is a repair enzyme that serves to keep its endogenous substrates at a very low level. To address this concern, we determined *Vmax* and *Km* values for the well-characterized methyl acceptor isoAsp-DSIP. Fig 5 shows raw data for A7P and I58V on a simple Vo vs [S] plot. The fitted curves were generated using the *Vmax* and *Km* values determined by the Direct Linear Plot method of Eisenthal and Cornish Bowden [17, 18]. A summary of *Vmax*, *Km*, and relative enzyme efficiencies are provided in Table 2. For both variants, the *Km* for isoAsp-DSIP was much higher than with WT, resulting in relative efficiencies of 34% for A7P and 60% for I58V. Although these two variants appear to be superior enzymes in the standard assay, they are almost certainly less effective for protein repair *in vivo*, as explained in the Discussion.

**Table 2:** Kinetic constants for methylation of isoAsp-DSIP by A7P, I58V, and F72L.

	γ-Globulin standard assay	isoAsp-DSIP			
Variant		*Vmax*	*Km*	*Vm/Km*	Relative efficiency
WT	100 ± 10	100 ± 12	7.4 ± 2.3	13.6	100
A7P	193 ± 17	200 ± 39	43.3 ± 10.8	4.6	34
WT	100 ± 10	100 ± 5.8	7.6 ± 0.8	13.2	100
I58V	188 ± 17	253 ± 52	32.2 ± 9.8	7.9	60
WT	100 ± 10	100 ± 2.5	9.4 ± 0.5	10.7	100
F72L	71.3 ± 5.8	66.0 ± 6.2	9.2 ± 1.8	7.2	67

γ-Globulin assay data, taken from our previous study, is included to facilitate comparison with the V*max* values obtained with isoAsp-DSIP. For γ-globulin, the standard deviation of triplicate assays is given. For isoAsp-DSIP assays, values are means and standard deviations obtained with the Eisenthal/Cornish-Bowden Direct Linear Plot calculations. All V*max* values are normalized to the mean value of WT PIMT samples done in parallel.

We also carried out a kinetic analysis of F72L. This variant has the highest allele frequency of all the rare human PIMT variants known to date, and was the third most damaging SNP in our previous study, exhibiting an activity loss of about 30% relative to WT. Given its high allele frequency, we were interested to see if it’s *Km* for isoAsp-DSIP was also affected. As shown in Table 2, the *Km* is indistinguishable from that of WT, indicating that this mutation affects catalytic turnover, but not isoaspartyl substrate binding.

## Discussion

### Four new SNPs with functional deficits

In our previous study of 13 rare missense SNP variants of human PIMT, we called attention to four: R36C with less than 1% activity in our standard assay, G175R and F72L with activity losses of 65% and 30% respectively, and R17H with highly significant thermal instability. Among the four new variants in the current study, all showed significant deficits relative to WT PIMT. L191S was by far the most affected in all three categories of assessment (Figs 2–4). A150V and P174H showed activity losses almost as great as L191S, and levels of aggregation higher that of wild-type PIMT, but differ significantly with regard to thermal stability. A150V showed a large decrease in thermal stability, while P174H exhibited a slight increase relative to WT. A65V, the least affected of the four, showed a modest loss of activity, a significant loss of thermal stability, and no difference from WT in aggregation.

### Reliability of SNP effect algorithms

The functional damage seen in all four of the newly expressed variants seems to justify our use of SNP-effect algorithms to select these variants from a larger set. If we look back at how these algorithms predicted effects for our previous set of variants (lower section of Table 3), we see a good correlation. R36C and G175R are justifiably tagged for very low activity in the standard assay, while A7P and I58V were justifiably tagged for their low efficiency. Two other previous variants with less severe deficits (R17H and F72L) were not tagged by any of the three algorithms, although the fathmm algorithm did come close with R17H. These algorithms depend in part on the assumption that mutations at highly conserved sites have a high probability of disrupting function. The high success rate with human PIMT prediction likely stems from the fact that PIMT is present in a wide range of organisms whose genomes have been sequenced, providing a strong basis for comparisons.

**Table 3:** Summary of PIMT variant damage observed in the current study, and our previous study.

Variant	Relative Activity^a^	Relative Efficiency^a^	ΔMP(°C)^b^	Relative Aggregation	Damage Predictions		
					SIFT	fathmm	PP-2
**Current study**							
A65V	0.89 ↓		-3.4 ↓↓↓	-	0.00	-4.59	1.00
A150V	0.36 ↓↓		-4.5 ↓↓↓	↑↑	0.08	-4.23	0.78
P174H	0.39 ↓↓		+0.5↑	↑↑	0.04	-5.40	0.54
L191S	0.28 ↓↓		-5.2 ↓↓↓	↑↑↑	0.01	-3.10	1.00
**Variants from our previous study**							
A1V	1.00		-0.6 ↓	-			
A7P	1.93 ↑↑	0.34 ↓↓	-1.3 ↓↓	-	0.14	-2.38	0.47
R17H	1.00		-5.0 ↓↓↓	-	0.12	-2.81	0.68
A33S	1.00		-2.4 ↓↓	↑	0.53	-1.09	0.00
R36C	<0.01 ↓↓↓		-2.0 ↓↓	↑	0.00	-9.18	1.00
H38N	1.00		-2.6 ↓↓	-	0.32	-1.96	0.00
I58V	1.88 ↑↑	0.60 ↓	-1.3 ↓↓	-	0.31	-2.91	1.00
F72L	0.72 ↓	0.67 ↓	+0.3 ↑	↑	0.37	0.38	0.00
A79G	1.00		-2.6 ↓↓	↑↑	0.33	-2.22	0.00
V119I	1.00		+1.6 ↑↑	↑	0.36	0.04	0.00
K124R	1.00		-0.2 ↓	-	0.31	-0.96	0.01
P162S	1.00		-0.1 ↓	-	0.37	-1.42	0.01
G175R	0.55 ↓↓		n.d.	↑↑↑	0.06	-7.57	1.00

Colors indicate probability that the mutation is damaging: red (high), tan (moderate), clear (low), as predicted by the indicated algorithm. Arrows provide a qualitative indication of change relative to wild type.

^**a**^ Enzyme Activity (standard assay) and Efficiency (Vm/Km) are relative to WT = 1.00.

^**b**^MP: change in Melting Point by DSF.

### Catalytic efficiencies of A7P, I58V, and F72L

We carried out kinetic studies on A7P and I58V in the hope of establishing the significance of their unusually high specific activity, about twice that of WT as measured in our standard assay. We found that both variants suffer from a large increase in *Km* for isoAsp-DSIP, indicating they are less effective at repairing isoAsp-damaged proteins under *in vivo* conditions wherein steady-state levels of such substrates are expected to be very low. If we look at relative rates at 5 μM isoAsp-DSIP in Fig 5, it is seen that the rate reduction for A7P is substantial (~50%), whereas the reduction for I58V is quite modest owing to its very high Vmax. A7P and I58V illustrate a trade-off that must be made in the design of enzyme active sites: selective binding of substrates *vs* optimal alignment for the transition state. It appears that, in these two variants, the active site structure has shifted in favor of transition state accommodation that increases the turnover rate, but at the expense of weaker substrate binding.

In addition to A7P and I58V, we determined kinetic constants for F72L, which exhibits a 30% loss of activity in the standard essay. We wanted to see if its enzyme efficiency was even lower, because F72L has the highest allele frequency of any rare PIMT variant found to date. As shown in Table 2, F72L has the same *Km* for isoAsp-DSIP as WT, indicating that this mutation affects turnover rate, but not isoaspartyl substrate binding.

### Artificial mutagenesis at Arg-36

We previously found that the R36C variant of PIMT has that less than 1% the methyltransferase activity of WT, leading to speculation that the Arg guanidino nitrogens might be essential for binding of AdoMet *via* interaction with its methionyl α-carboxyl, a proposal consistent with the 3D structure of human PIMT with AdoHcy bound (PDB: 1I1N). We tested this idea by assaying the activity of two artificial variants, R36A and R36K. As predicted, the R36K variant does have more activity than R36C, although the restoration was modest, possibly due to a longer distance and suboptimal geometry of the putative charge-charge interaction. The complete lack of catalytic improvement in R36A implies it is the absence of the Arg side chain that is destructive, rather than some unique interference of the active site by the cysteine side chain. Interestingly, a similar type of mutation, R49C in human S-adenosylhomocysteine hydrolase (AHCY), reduces its catalytic activity to 6.7% of WT [19].

### Medical implications

As noted in the Introduction, and in our previous paper, there is substantial evidence that absence of PIMT activity in mice results in severe neurological problems, including fatal epilepsy, while moderate variations in PIMT activity in humans appear to affect longevity and the quality of life, both cognitive and physical. A qualitative summary of the damage associated with each rare PIMT SNP from both our studies is presented in Table 3. If we focus on enzyme activity and/or efficiency, the most deleterious mutations are A150V, P174H, and L191S from the current study, and A7P, R36C, I58V, F72L, and G175R from our previous study.

The population frequency of these mutations is shown in the top half of Table 4; the bottom half shows additional new variants that have a high probability of damage as predicted by at least one of the algorithms we have used here, but have not yet been studied. R36C, by far the most severe mutation, was found in a study of Europeans at a frequency corresponding to 1 heterozygote per 2,250 individuals (4,300 alleles). F72L, is the most frequent of the “rare” mutations reported to date, was found exclusively in the ExAc European/Finish population with a frequency corresponding to 1 heterozygote per 606 individuals. A single case of P174H was found in the east Asian population, while 2 cases of I58V were found in the south Asian population.

**Table 4:** Population frequency of PIMT variants with observed or suspected functional damage.

			Global MAFx100	Ethnic or racial group^a^						
Variant	Source	PosX		AFC	EUR	FIN	LAT	EAS	SAS	OTH
***Variants with observed activity loss in this and our previous study***										
A7P	dbSNP	66	0.019246							
R36C	GO-ESP	95	0.015378	0/4,406	**2/8,600**					
I58V	ExAC	117	0.013870	0/564	0/4,066	0/1,596	0/188	0/198	**2/7,620**	0/184
"	GO-ESP	117	0.007689	**1/4,406**	0/8,600					
A65V	ExAC	124	0.000834	**1/10,400**	0/66,392	0/6,614	0/11,562	0/8,654	0/15,462	0/892
F72L	ExAC	131	0.046230	0/10,406	**55/66,656**	**1/6,614**	0/11,578	0/8,654	0/16,308	0/906
"	GO-ESP	131	0.030755	0/4,406	**4/8,600**					
A150V	dbSNP	209	0.000824							
P174H	ExAC	233	0.000827	0/10,256	0/66,576	0/6,606	0/11,560	**1/8,618**	0/16,448	0/906
G175R	ExAC	234	0.004955	0/10,306	**4/66,614**	0/6,606	0/11,570	0/8,622	0/16,454	0/906
"	GO-ESP	234	0.007689	0/4,406	**1/8,600**					
L191S	dbSNP	250								
***Newly reported variants with predicted defects***										
V30M	ExAC	89	0.000829	0/10,262	**1/66,306**	0/6,614	1/11,524	0/8,628	0/16,448	0/902
H38R	ExAC	97								
I52L	ExAC	111	0.000829	0/10,266	**1/66,288**	0/6,610	0/11,510	0/8,618	0/16,446	0/902
I52V	ExAC	111	0.000829	0/10,266	**1/66,288**	0/6,610	0/11,510	0/8,618	0/16,446	0/902
G53D	ExAC	112	0.006935	0/564	**1/4,070**	0/1,596	0/188	0/198	0/7,620	0/184
K80E	ExAC	139	0.000825	0/10,406	**1/66,686**	0/6,614	0/11,578	0/8,654	0/16,414	0/908
I106T	ExAC	165	0.005851	0/10,324	**1/66,200**	0/6,606	0/11,544	0/8,634	0/15,446	0/892
I108L	ExAC	167	0.000834	0/10,336	**1/66,274**	0/6,606	0/11,546	0/8,638	0/15,686	0/896
V139M	ExAC	198	0.000833	0/10,274	**1/65,896**	0/6,598	0/11,486	0/8,606	0/16,228	0/900
A167V	ExAC	226	0.001648	0/10,404	**1/66,704**	0/6,614	0/11,576	0/8,650	**1/16,506**	0/908
V182L	ExAC	241	0.000824	0/10,388	**1/66,706**	0/6,614	0/11,578	0/8,650	0/16,492	0/908

For each ethnic group, frequencies greater than zero are high-lighted in bold face. Each of the “newly reported variants” listed in the bottom half of the table have a high probability of damage predicted by at least one of the three SNP effect algorithms.

^a^Based on abbreviations used by ExAC: AFC, African or African-American; EUR, European (non-Finish); FIN, Finish; LAT, Hispanic/Latino, EAS, East Asian; SAS, South Asian; OTH, Other.

It should be noted that entries in all the SNP databases we used in this study are subject to errors. For example, a study published in 2004 [20] claimed a false positive rate of 15–17% in dbSNP, though with improving technology and technique, error rates are falling to lower levels [21]. With this in mind, we caution that there is some uncertainty about the validity of the frequency data shown in Table 4. We are most concerned about A7P, A150V, and L191S due to the lack of detail provided by dbSNP and the fact that these variants are not present in the ExAC database.

**Fig 6:**
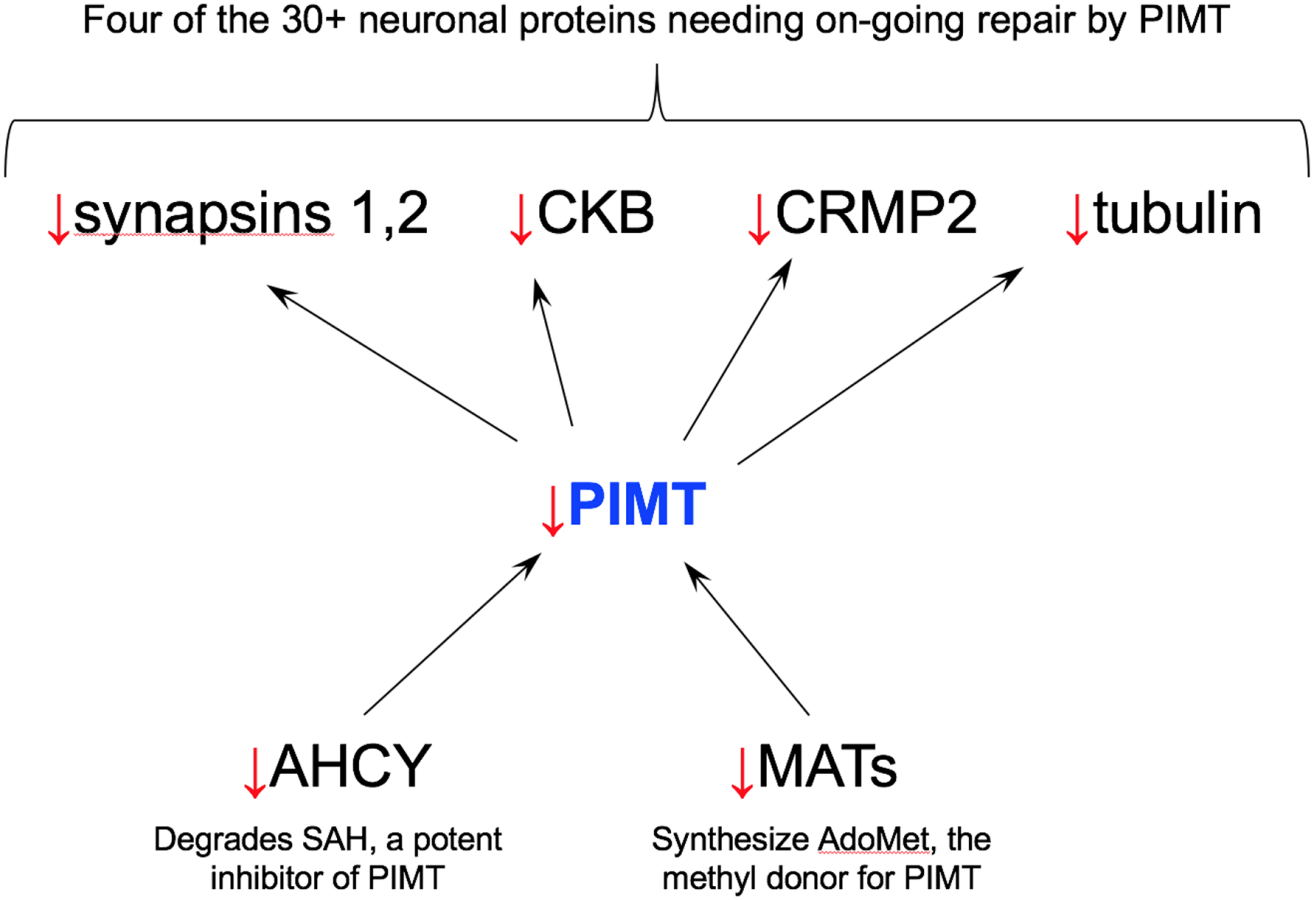
Illustration of interactions by which PIMT deficits could amplify the effects of deficits in other proteins. Abbreviations: CKB (creatine kinase B), CRMP2 (collapsin response mediator protein 2), AHCY (S-adenosyl-L-homocysteine hydrolase), MATs (methionine adenosyl transferases). The PIMT repair targets shown in this diagram are among those identified by Zhu et al. [23] *via* proteomic analysis of brain extracts from PIMT KO mice.

Unlike the many enzymes, PIMT’s repair function renders it inherently pleiotropic, providing the basis for deleterious synergisms between harmful mutations in PIMT and harmful mutations in the numerous proteins with which it interacts. An illustration of such potential interactions is shown in Fig 6. Reduction in AHCY activity will slow down rates of protein repair by allowing accumulation S-adenosyl-homocysteine, a potent inhibitor of PIMT. Extreme reduction of AHCY by itself leads to severe developmental problems and early death in children [19]. Mutations in AdoMet synthetases (MATs) will decrease the availability of the methyl donor required for PIMT-dependent protein repair. Deleterious synergism should also occur between mutations in PIMT and any of the numerous isoAsp-prone proteins that require ongoing repair. These negative synergisms are not theoretical. In PIMT KO mice (prior to their death), the activity of brain creatine kinase (CKB) is reduced by 30%, coincident with accumulation of isoAsp sites in this enzyme [22]. In PIMT HZ mice, isoAsp-damaged proteins accumulate 3–6 times faster than WT mice as they age [8].

Epilepsy, the dominant phenotype of the PIMT-KO mouse, is present in a significant sub-population of children diagnosed with autism spectrum disorder, a disease notorious for its genetic complexity. To our knowledge, there are as yet no known PIMT SNPs with a strong association to autism, epilepsy, or any other neurological disorder; although we note here that the ExAc database states it is devoid of data from “… individuals with severe pediatric disease” (exac.broadinstitute.org/about). As disease-specific genome sequencing advances in scope, we feel confident that deleterious PIMT variants, several of which are described here, will arise as contributors to one or more neurological diseases in the young, and to progressive CNS disorders in the aged.
